# Get this thing out of my body! Factors determining consent for translational oncology research: a qualitative research

**DOI:** 10.1186/s12967-023-04039-0

**Published:** 2023-05-21

**Authors:** Desprès Caroline, Mamzer Marie-France

**Affiliations:** Centre de Recherche des Cordeliers, Sorbonne Université, Université Paris Cité, Inserm, Laboratoire Êtres, Unité Fonctionnelle d’Éthique Médicale, Hôpital Necker-Enfants Malades, APHP, Paris, France

**Keywords:** Biobank, Consent models, Informed consent, Broad consent, Translational research, Trust

## Abstract

**Background:**

Depending on the needs of scientific research at a given time, biobanks make biological samples and data available to researchers. In this article, we aim to describe the reasons and underlying logic that determine the decision to grant or deny consent to the conservation of tumour samples in a biological resource platform for research purposes. We make use of the CARPEM biological resource platform model, where broad consent is required.

**Methods:**

The results are based on semi-structured interviews, conducted between 2019 and 2021, with 25 individuals having various profiles.

**Results:**

All the people interviewed readily accepted the principle of conserving a tumour sample for research purposes. They explained their decision by citing the desire to participate in research dedicated to improving therapeutic medicine. Their trust in research institutions or in doctors was an important factor in their consent. The tumorous nature of the samples also played an important role, as did the absence of constraints. Finally, the high level of consent was also based on the difficulty they had in conceiving what the future risks might be once the sample had been taken, whereas the fact that they did not know the nature or purpose of the research to be carried out when they signed the consent form posed some problems. These results stem from a lack of a culture of ethics among the people interviewed.

**Conclusion:**

The information provided in the context of consent at the CARPEM tumour bank seems inadequate for consent to be considered 'informed', given the low level of knowledge that people have of the risks and issues. Information is missing even though we feel it would not change consent or only marginally. This raises questions, since part of the act of granting consent is based on the implicit trust French people have in the hospital that collects the data and in research practices in general. In the minds of those who participate, transparency is the ground on which trust rests. Lack of transparency could be deleterious for future research practices. However, it is not by striving to improve information leaflets that the consent-related information will improve but, rather, by more effectively helping future patients to assimilate that information.

## Introduction

Access to human biological samples and data is increasingly important as research in the field of translational medicine continues to develop. Thanks to biobanks [1], significant quantities of such samples and data are readily available, whatever the needs of scientific research may be at a given time.

The sampling of cells from human tumours is an invasive procedure usually undertaken for diagnostic and theranostic purposes in the context of a patient's healthcare. In France, it is a regulated obligation to conserve the samples so that a diagnosis can be confirmed if necessary. They are also of potential interest for researchers, and the repurposing of the samples is thus becoming increasingly important for medical progress.

The regulatory framework for the use of human biological products for clinical or translational research purposes requires consent on the part of the patient. The various national and international laws and regulations establish—explicitly or implicitly—a strong link between information and consent, so-called 'informed consent'. Specific ethical questions thus arise with biobanks insofar as the uses, means and technologies available for analysis and the research questions that will arise in the near or distant future are impossible to predict: "[A] scientific context is always being renegotiated and is never predetermined" [2].

Explicit consent (or opt-in) has become common practice for integrating biological samples and data into biological resource banks. This is not mandatory under French law, which only requires that the patient be informed and expresses no opposition. Rather, this practice results from international collaboration: French researchers share their resources and publish in international journals whose rules of good practice require explicit consent.

The specific consent model, traditionally used in clinical trials, is ill-suited to new forms of research, particularly when it comes to collecting biological samples. It is costly and time-consuming, and not very effective: people who must be solicited again after an initial consent are virtually impossible to recontact [3, 4]. In contrast, broad consent allows flexibility and rapid access to samples and data in response to emerging research needs. It also poses fewer economic and logistical problems and is widely preferred by researchers. However, it constitutes a threat to individual autonomy and trust. There is little research devoted specifically to analysing the distribution of the different forms of consent used for translational research, but a recent French study shows that for the translational cancer research consortium SIRIC CARPEM, broad consent is the more common of the two [5]. In this context, the traditional notion of informed consent needs to be reconsidered. The question arises as to whether consenting to an unknown future use is an expression of will and whether it can truly be called informed.

Dynamic consent allows for more active participation of the patients who provide samples and data and gives them access to real-time information about ongoing research. It also affords patients the possibility to withdraw consent at any time [4]. Although this was first presented as an ideal solution, the literature on the subject has become increasingly critical. Steinbeck [6], for example, insists that the difference between broad consent and dynamic consent is not so clear-cut, that biobanks are increasingly required to provide information to their donors and that too much information can be problematic and even self-defeating. The goal, rather, should be to provide information that is relevant and understandable [7, 8]. Recently, Mikkelsen et al. [9] argued than even it is not a perfect solution, broad consent is best suited to deliver participant protection while achieving the research aims of the biobank.

In the collection of human biological samples, the willingness of individuals to consent varies from one study to another. It depends on variables such as ethnic minority status (USA), level of education, household income, employment status and socio-demographic or socio-economic factors. In these studies, acceptance rates generally vary according to the different forms of consent [4] whereas in a more recent study, Sanderson [10] shows that they ultimately have little effect on the inclination to consent. This varies according to national and local contexts [11] and also depends on the organisations (public versus private) with which these resources are shared and on the objectives, especially if commercial use is potentially involved [12]. Nevertheless, the rate of consent is generally high [13]. Donors' motivations include contributing to medical progress in general (especially for future generations [11]), or more specifically to fighting disease. They also include improving treatments in order to 'save lives'. The nature of the research carried out may give rise to reservations related to donors' personal values, to their religious, moral or philosophical beliefs [14] or to the risks incurred, especially when these involve invasion of privacy, violation of medical confidentiality or risks of discrimination.

The CARPEM (CAncer Research for PErsonalized Medicine) project is based on a consortium of French health care and research teams, supported by translational research platforms in the framework of international cooperation. It has a biological resource platform (or BRP) including a tumour bank.

The tumour samples in the biobank are taken either as part of a diagnostic biopsy or, more often, during a surgical procedure to resect a malignant tumour or a diseased organ for therapeutic purposes. Cross-sections are then made and some of them are cryo-frozen. No further action is required from the patient. In this context, broad consent is the form of consent used and the type of research to be carried out is not known. Only the scope is specified—in this case, cancer research.

In this article, we aim to describe the reasons and logic behind a patient's decision to grant or deny consent to the conservation of tumour samples in a biological resource platform for research purposes based on the CARPEM biological resource platform model. Beyond the explicit reasons given by individuals to motivate their consent, we are interested in the individual and/or social logics that govern their willingness to consent to the re-use of samples and data for research purposes. In the context of a biobank, and more specifically a tumour bank, we examine whether the manner in which consent is solicited corresponds to current ethical principles.

## Methodology

### Analysis of the way in which information is provided and consent solicited

Our approach involved meeting with several professionals from the BRP: 7 clinical doctors who solicit patients' consent on a daily basis, and the secretarial offices of 9 hospital wards whose tasks involve having consent forms signed.

The fact that another research project on breast cancer was being conducted at the same time gave us the opportunity to observe first-hand how information was provided and consent solicited in a single hospital ward. More specifically, we observed ten pre-surgical consultations in order to better understand the concrete context of soliciting consent and how procedures differ from one doctor to another. Interviews with doctors from other wards enabled us to broaden the scope of the practices observed.

### Patient interviews

Our results are based on 25 individual qualitative semi-structured interviews with several categories of people carried out in 2019 and 2021. Eleven of them were treated in the hospital in question for cancer or high risk of cancer (Lynch syndrome) and 3 were treated in another hospital. The other 11 participants were not cancer patients; they were either (1) data experts and researchers in the fields of IT, law or health, or (2) laypeople with no particular knowledge of these fields (see Table 1). The goal of these interviews was to analyse the factors which determined their participation in a biobank and to evaluate their knowledge and expectations in terms of research ethics. Our study population was therefore made up mainly of people who had not been confronted with a request to preserve their samples and who therefore were not yet familiar with the consent form used by the PRB.

**Table 1:** Characteristics of the people interviewed

Male/female	Age	Profile	Location	Means of recruitment
F	68	Artist Nièvre	HEGP^a^	Breast cancer
F	50	Flight attendant	HEGP	Breast cancer
F	45	Consultant Chinese/92 Refusal of radiotherapy	St Jo	Breast cancer
F	42	In nursing training	HEGP Consultation for Lynch Syndrome	Genetic predisposition
M	55	Engineer	HEGP Consultation for Lynch Syndrome	Genetic predisposition Daughter deceased
M	77	Inspector General of a government department/retired	HEGP Consultation for Lynch Syndrome	Polyposis in the family
M	46	Telephone sales technician, Algerian-French	HEGP Consultation for Lynch Syndrome	Genetic mutation + Cancer
M	29	Librarian	HEGP Consultation for Lynch Syndrome	Genetic mutation
F	64	Military Armed Forces Medical Service	HEGP Consultation for Lynch Syndrome	Colon cancer + Genetic mutation
F	57	Early retirement (housewife) Lives off investments	HEGP Consultation for Lynch Syndrome	Genetic mutation
F	77	Retired	HEGP Consultation for Lynch Syndrome	Several cancers Genetic predisposition

"Expert patients" with cancer			
M	65	Expert patient	Laryngeal cancer
M	50	Expert patient	Intestinal cancer
F	42	Expert patient	Breast cancer
F	55	Head of Association	Parent of a child with cancer

Computer/data/data mining/AI experts = 10 people			
M	29	Artist and programming engineer	
M	53	Banker	
F	32	Artist Ph.D. student in AI and artistic practice	Mother died of cancer
F	55	Researcher in Health Economics	Public (academic)
F	29	Legal Officer	Start up on health ethics
F	48	Researcher in health economics	Private/worked in a cancer centre
F	40	Researcher in health geography	Academic
F	34	Doctoral student in Philosophy	Academic
M	35	Philosopher of Technology	Academic
M	24	Data analyst	Private university structure

Group of people living in poverty: 10 people					
Sex	Age	Gender	Social situation	Living situation	Specific situation
F	62	F	RSA^b^	Lives alone	Helps migrants
F	65	F	Retired	Lives alone	Helps people in difficulty
F	66	F	Retired	Lives alone	
M	58	F	AAH^c^	Lives alone	Disability Under tutelage Heart problems
M	67	H	Retired	Married with children	
F	56	F	AAH	Married with children	Motor disability
F	68	F	Retired	Lives alone	
F	69	F	Retired	Lives alone	
M	64	H	Retired	Shelter	
F	64	F	AAH	Shelter	History of cancer

^a^Hôpital Européen Georges-Pompidou

^b^The *Revenu de Solidarité Active* is a French welfare benefit aimed at reducing the barrier to return to work

^c^The *Allocation aux Adultes Handicapés* is a French welfare benefit for disabled adults

In addition, two group interviews, 6 months apart, were conducted with a specific group of people living in extreme poverty, a profile rarely consulted in studies. This group was made up of ten people involved in ATD Fourth World. The first interview was designed to gather opinions on the sharing of health data for biomedical research, to analyse their willingness to consent to the repurposing of tumour samples and to evaluate their perception of the risks. The second interview explored their understanding of the consent form used by the tumoral resources platform.

The socio-anthropological analysis that resulted from these interviews went beyond the classic themes of the factors determining consent by including the symbolic dimensions associated with tumour biospecimens and the social meanings of consent.

## Results

### Attitudes towards and motivation for granting consent

From the outset, all the people we interviewed accepted the principle of conserving tumour samples for research purposes as part of a broad consent. Not knowing the nature or purpose of the research that would be carried out when they signed the consent form did not alter their attitudes.

For two-thirds of the people interviewed, the consent given was hypothetical. It was clearly explained before the interview was conducted that this hypothetical situation was taking place in a hospital setting. The doctors told us that this same information was given to the people they explicitly asked to participate in the tumour bank during a medical consultation. The rate of refusal reported by the BRP was very low.

#### Contributing to cancer research

The reasons given for consenting to research on the samples and/or data revolve around participating in the future of scientific progress: 'It can save lives'.

Well, if it can help science progress a little, it's a good thing. (male, age 29, librarian, predisposition to cancer)

*This would allow research to be carried out on it. To advance research, to better understand the development of the pathology. Potentially to promote the reimbursement of medicines and not only that. (female, age 40, geographer)*

Participation in research was described by some as 'natural' or 'normal'. These words came up repeatedly, and in the mouths of different people. For them, it was logical and self-evident. They did not even need to think about it.

*I wouldn't even ask myself the question. It seems obvious to me. (female, age 40, geographer)*

The question even surprised some of the people interviewed, and this led us to ask them if they deemed it necessary for their consent to be obtained. This question was above all a way of getting them to talk about their ethical expectations and needs. We will come back to this point later.

It is therefore most often a question of contributing to the 'progress' of science and medicine, demonstrating an altruistic and disinterested perspective, without any expectation of personal benefit; sometimes it is a question of showing solidarity with, or even responsibility towards, future generations.

### The nature and value of the samples

We wanted to know the extent to which the nature of the biological sample might be related to the willingness of individuals to consent to the conservation of their tumorous samples and data for future scientific use.

The most popular representation of cancer is that it is an exogenous disease [15]. It is frequent to hear patients say that they want to get rid of what they consider to be an 'intruder' (female, age 34, doctoral student in philosophy) and something alien:

*I think for all of us it's a kind of foreign body that develops in us, so we want to get rid of it. So for me, no reservations. (male, age 50, expert patient)*

*If I have a tumour, I just want it removed and I don't want to see it anymore. [...] Get this thing out of my body and if you want to use it because it's useful for you, fine! (female, age 32, artist)*

The exact opposite representation of cancer is that it is a disease emanating from the patients themselves, an anarchic proliferation from their own body. This representation is closer to our biological and genetic knowledge on cancer. The words 'it's a piece of oneself' came up regularly:

*Because it's us, it's a part of us. I mean, there's a part of our being that's in there anyway. Even if it's useless, even if they're cancerous cells, even if we want to get rid of them, we're at the origin of it all the same... (female, age 42, patient-expert, breast cancer)*

*It's made up of cells that are mine. (female, age 34, doctoral student in philosophy)*

These two representations sometimes coexist: an internal dysfunction, the origin of which is still unknown but which produces an anarchic proliferation that must be eradicated and torn away from the patient.

*Since it's something malignant that has to be removed, it's not exactly a part of me. Yes. Yes, that's a bit how I feel, because it's malignant (female, age 42, breast cancer)*

*It is a part of me, it is made up of cells that are mine. But it shouldn’t remain there. It shouldn't be there in the first place. There's a bit of a contradiction between these two things. (female, age 34, doctoral student in philosophy)*

Moreover, in contemporary Western societies [16], once bodily substances are removed and physically detached from the body of the person, they are no longer living. They change their status, in much the same way as a body becomes a corpse after death. Once taken, the sample is separated from the body physically and symbolically: it becomes an object whose future we are no longer interested in and that ultimately disappears.

*Does this sample taken belong to me? Once it is taken? No, there's no problem. I won't ask for it back! (laughing) (female, age 42, nurse in training)*

This process of 'detachment' is reinforced by its tumoral nature. What comes to the fore is the desire to *get rid of* these samples as quickly as possible.

*A piece of me, a tumour, well it's something I want to get rid of, that's it. I don't think there is any problem in signing this consent if it can help research. I have no qualms about getting rid of these tumour cells. (male, age 29, librarian, predisposition to cancer)*

This perception needs to be qualified. The interviews show that not all samples have the same value to people: the resection of a tumour and the removal of a diseased organ (or part of an organ), part of which will be frozen and preserved, are two different things. Some samples carry the identity of the person and have more symbolic value as in the case of gametes [17]. All these samples carry the patient's genetic heritage, but they differ in the image or representation that they convey and in their implied potential uses.

For example, some women ask their doctors what will become of the diseased organ that has been surgically removed. Thus, there is a significant difference in people's perspective depending on whether it is a tumour that is being removed or an organ (breast, uterus, ovary, colon or lung, for example), even if the organ is contaminated by cancer.

When an organ has a strong symbolic value, it is not because it has been removed that it becomes simple waste. Thus, parting from and getting rid of it is not trivial and sometimes requires reflection. For some people, even if in most cases they do not ask what will become of it, the thought that an organ will end up in a trash bin is unnerving. One woman who had a breast tumour removed explained that if it were a whole breast, she would think twice before giving her consent.

*Let's say that when it's just a piece, it's not the same thing as when it's the whole organ (female, age 42, breast cancer)*

### Data representation

The way people relate to the health data that are associated with samples is quite different. These data are more difficult to understand, and some people find it hard to apprehend their meaning given that they have no substance. They are not only immaterial, they are also abstract, contrary to samples, which are 'corporeal' and therefore 'tangible' (female, age 34, doctoral student in philosophy). Health data are diverse. They take the form of text (words and figures) or images and are often the result of a technical device and/or of a professional's interpretation. They generally give an account of biological facts, of which they are a representation, or provide information on a family or social situation.

The majority of people did not realise that, the personal health data collected while they were in hospital could be used for research, unless they objected. Some of them had no idea that their biological samples, to which they had consented for use in research, could be linked with their health data. Once this was clarified, the vast majority of them felt exposed to risks concerning medical secrecy and their privacy.

As long as anonymity has been guaranteed through the de-identification of the sample, people are generally considered to be protected from these risks. However, fears do persist, particularly those centered on the figure of the 'hacker': people knows that it is impossible to make data of this type totally secure, since they will in any case be shared and made available to others.

### The representation^1^ of risk and expectations

#### A representation of risk based on the experimentation model

For a majority of the people in this study, the conception of the risks associated with their participation in medical research was limited. Take, for example, this woman (age 55, economist), who considered that since the sample had already been taken, the risks were behind her. She felt little concern about the use that could be made of it:

*In fact, the thing has already been taken away from me, so it's already been done. It would be more the risks for me than the fact that my marrow might be put to bad use, for example. For me it would be that. I would be more afraid of the consequences for me.*

Patients or non-patients, experts or non-experts, once the sample has been taken, they hardly imagine that there might be risks, their definition of a risk being that it is something life-threatening or at least physically damaging.

*What's the downside of this? I don't see the problem. (male, retired, genetic predisposition)*

*About the samples, I have no… they go to the laboratory, it's not… About the risks, frankly, I don't see what they are. (female, age 42, nurse training)*

#### The (non-)representation of the uses of samples and data

Moreover, people generally have a rather vague vision of the use that will be made of these samples. At the same time, the specific context of research limited to cancer accounts for these results:

*I don't feel that with my blood sample they can do anything dangerous. Or that if I had a mole or a tumour, they could do dangerous things with it. (female, age 55, economist)*

One might imagine that patients anticipating a potential misuse of their samples would request a right of review concerning the research to be carried out and the personal values they want to be respected. A number of them, however, had a somewhat distorted idea of what such misuses would actually involve, mentioning situations that do not really fall within the scope of research conducted by the CARPEM consortium. These included malicious use outside the field of research such as data theft or the sharing of data with entities that could make non-scientific use of them, or even manipulate them genetically.

*You can imagine anything that has to do with genetics, cross-breeding and using cells to create other things. (male, age 55, engineer)*

As for the experts in our study population, they were not very worried about misuse or malicious use. Only those who anticipated misuse were more likely to request a right of review concerning the research carried out and the values that they wanted to be respected.

#### Anonymisation

For most people, anonymisation is a condition of consent in order to preserve privacy. Some, however, consider that 'they have nothing to hide' and attach no importance to anonymity:

*I don't care if my tumour has my name on it or not. (female, age 57, genetic predisposition)*

Although on the whole our interviewees expressed few concerns about the anonymisation of data, this does not mean that this issue was unimportant for them. One man working in the telephone industry recalled the need for anonymity 'so that individuals cannot be traced' while at the same time recognising the importance of being able to contact a person should a predisposition be discovered (which was his case). More often than not, the people interviewed were not aware that these samples were linked to personal data and that the analyses carried out would in turn be transformed into data. Moreover, they assumed that researchers and doctors would ensure that the rules concerning the protection of the data resulting from the analysis of the sample or linked to it would be respected.

*And so anonymity is kind of the basic principle for you to give your consent! (male, age 29, librarian, genetic predisposition)*

Among those who said they did not attach much importance to anonymity, they assumed that circulation of this information was restricted to the research community:

*Explain to me what the risks are. That they will tell people that so-and-so has some shameful disease? What will they say next? It's all anonymised, I suppose. (male, age 77, retired)*

*I don't see how this information could be harmful to me personally as long as it is not given to my insurer or my banker. Or to my employer. That's three people. (female, age 55, economist)*

Sometimes they imagined that the sample would be used only at the hospital where it was taken.

*No. Maybe I have so little to hide that... no, no, I don't see what... I don't see any risk. [...] But normally, it doesn't leave the hospital or laboratory, right? (male, age 29, librarian, genetic predisposition)*

Our interviewees also frequently pointed out that their individualised health data would be of little interest, which is also an argument for allowing access to commercial firms (connected health data, for example). Thus, individual data are seen as only making sense when analysed as part of a multitude of data, and individual data are always lost in 'a gigantic mass of data'.

#### Trust

The significant number of individuals who grant their consent is due to the trust they have in their doctor and more generally in research institutions. It is part of the positive representations of the figure of the 'expert', the doctor or doctor-researcher who obtains consent. This trust is extended to the institution. People assume that the system protects every citizen and every patient, and therefore that the protections are effective and guaranteed in the specific context of biomedical research. This is underpinned by a representation of the existence of a legal context and a model of governance.

*There are rules, there are ethics, there are… how can I put it… institutes set up for this, which are well established, which really… (female, age 50, genetic predisposition)*

In people's minds, rules of this kind cover several fields, such as protection of the life of individuals and the proper use of data.

For data experts, the risks are not necessarily significant in the context of research and more specifically biobanks, provided that certain guarantees are respected. The knowledge of the processes, particularly that of de-identification, especially reassures those experts who know these processes better.

*I think I have a naive belief in the benevolence of researchers. And of the structures that stock data for research purposes. I don't believe that these people are malevolent. But I think that when I go to a hospital, it doesn't have the same effect on me because it's an institution. [...] I don't mind if a university hospital does this to me without asking me any questions... (female, age 55, economist)*

Finally, we noted a greater distrust of hospitals or research institutions coming from people in precarious situations, although it did not prevent them from granting their consent. Their greatest fear was that data relating to their social status and lifestyle might be disclosed to the medical team or the social workers helping them. While they were rather willing to participate, sometimes without any restriction, one of them demanded confidentiality. In his personal experience, medical secrecy had once been betrayed by his doctor, which led him to distrust researchers.

*I won't be satisfied with words saying 'we can assure you'. No! An official written document; that’s what I need, a sort of, I don't know, a notarised document... In that case, yes, I can consent. (male, age 64, activist)*

Trust in research organisations and hospitals does not extend to all institutions and sometimes not to public authorities. It does not extend at all to pharmaceutical companies. It was necessary to explain to participants that their data could be shared with external organisations or institutions. The purpose of the biobank is to share data with multiple research institutions, and not just local university hospital teams. This policy of shared data raised in some of them questions and doubts that they had not initially mentioned.

#### To solicit or not to solicit consent?

A majority of the interviewees anticipated few ethical risks surrounding the conservation of biological samples for research, so it is understandable that some did not see the point in their consent being solicited at all. While the majority of them wanted their consent to be solicited, some were prepared to forego it. Take, for example, this man, who had intestinal cancer and a genetic predisposition to colorectal cancer:

*In my opinion, there is no need. (male, age 46, engineer)*

In view of the importance of advancing research, he would consent without reservation in order to save researchers time and resources. Several people thought that, as is the case for organ donation in France, it was not necessary to obtain consent, that is, presumed consent (opt-out).

*Personally, I'm really in favour of the fact that if there are data and if there is research to be done on my samples or whatever, for me there is no need for my consent. (female, age 56, activist, disabled)*

Others went so far as to advocate ignoring rules and procedures: if you wait for everyone's consent, you will never get anywhere.

*If we wait until everyone decides to say yes, research will never progress; we have to get that into our heads. It's anonymous data, so why should it bother us? It shouldn’t! (female, age 66, activist)*

The following woman was asked to sign a consent form in the same hospital, without being told the purpose and reasons for it. In this case, it was for a fluid sample rather than a tumour. She signed the document without reading it, even though she was shocked by the lack of information: why was she eligible, and what was the purpose of collecting this sample?

*Obviously, I'm for it and I'm for having the most representative biobank possible so that you can do research. I asked myself a 2*^*nd*^* question, I may agree to enter whatever cohort you want, but I first want to know which one and why. (female, age 55, economist)*

Although she was puzzled, she signed the form, thus losing some of her rights and giving up on her expectations.

For those who considered it important for doctors to solicit consent, we were interested in what it meant to the patients. Although the regulatory framework reassured them concerning past cases of misuse, most of them found it difficult to explain what they were.

*If they don't do it, even for people for whom it wouldn't be appropriate, it would kind of pave the way for future cases of misuse, in fact. (female, age 42, patient-expert)*

This woman, like others, insisted on the notion of respect.

*It's recognising the fact that the human body is something... It's something, how can I put it, it's a mechanism, it's a part of the physical world, but it also has a symbolic dimension, a very strong meaning behind it... A psychological one. It would mean denying all that, denying the person who... well, the patient, in fact.*

All this seems to mean that requiring consent is a matter of respect for human beings. More generally speaking, it is also connected to the question of the reification of the body, to the refusal by some to treat the body as a simple object or to depersonalise it [18], even if it is a dead body^2^ or a part of a body.

*That is to say, it is all about the attention we pay to another person's body, about recognising that it's not just matter, not just a body. [...] (male, age 50, patient-expert, intestinal cancer)*

*These are individual people, that's what we have to remember. It's not just any body, it's not just undifferentiated matter, it's him or her. There are individuals behind each body, and they have free will and an opinion on these questions. (female, age 42, expert patient)*

These words refer to the intrinsic moral value of consent: asking someone for their consent means treating them as a person.

Some see it as a matter of principle, a way of reminding people of the law:

*It's also, I think, a reminder that you can't just do whatever you want to do.*

*It's an ethical problem: as a matter of principle, we don't have the right to use someone without their permission. (male, age 65, expert patient)*

## Discussion

### A high rate of consent in a favourable context

Recall that in the context of translational research conducted within the framework of the CARPEM consortium, it is 'broad consent' that is solicited: the future use of the samples is not known. However, unlike some expandable biobanks, the research is limited to a single disease, which is cancer. The high number of people who agree to sign this consent form (both in our study population and as confirmed by the doctors we met) is necessarily related to this framework. These results can be found in the literature. They show that, in many countries, people agree to grant consent due to the fact that future research will be linked to the donor's pathology [19]. On this point, even people who were not cancer patients were willing to participate. Most people are not indifferent when it comes to cancer, which is considered to be the plague of modern society [20, 21]. This is true even today, despite the considerable progress made in the field of therapeutic medicine. Because it is still frequently linked to the image of death and suffering and to difficult, incapacitating forms of treatment, the disease continues to frighten people.

Furthermore, the fact that the samples are tumorous often results in people's being indifferent to what becomes of the samples or in their simply wanting to be rid of them, except when the sample in question is a whole organ with symbolic value. The sample first becomes worthless for two reasons: both because it has been severed from the living body and because it is cancerous. The repurposing of this surgical waste, the recuperation of it for new uses and the usefulness it can have for research reverses the process through a form of recycling. But giving it a second life can also become an argument against giving consent. In the literature, some women who have had an abortion refuse to 'donate' the foetus for embryonic stem-cell research, explaining that it would be as if their foetus would remain alive indefinitely [22]. More generally speaking, the new status conferred to the sample—now a useful or even precious resource [5]—could then change how people view future uses of it and the purposes and results of the research, without calling into question their willingness to consent.

The absence of constraints and of additional risks for the patient, insofar as the samples concerned were collected as part of their health care, helps to explain the high level of acceptance. This says nothing about what they would say if there were more constraints. The acceptance rate is lower when there is a constraint, such as additional tests, even as low-risk a test as a blood sample. Some participants in our study population refused to give their consent because of the travel and time it would involve.

In the absence of constraints, expectations concerning feedback are reduced. This explains why expectations regarding information on the uses that will be made of the sample and the data—and possibly the results of the research—are low. The more involved people are (depending on the specific purpose of the research) and the more active a role they play in terms of time and risk, the more they expect to be informed of the results and also of the use that might be made of them. It has to be worth it [19]: it is always the case for any just cause, such as advancing cancer research and—as participants frequently said—'saving lives', a rather vague promise with uncertain benefits. This promise of results [23] in terms of cancer care and treatment seems to unite people, since the vast majority are ready to participate. It is part of a certain form of optimism, the conviction that medicine will eventually overcome all diseases.

### A conducive—but ethically problematic—context for consent

A consent form including an information leaflet is provided to the patient, together with a brief individualised oral explanation, which each doctor provides in his or her own way. The different practices observed within the hospital depend in part on the degree of involvement of the heads of department and on the organisation and resources allocated for the process. In general, the information is given during a pre-operative consultation. Such a consultation, during which a great deal of essential information is transmitted to the patient, inevitably raises questions about the patient's ability to grasp what his or her consent is required for, and to understand what is at stake. Not only is the information provided orally and succinctly, but the consultation also occurs in an emotionally charged context whose aim is to define a course of care and sometimes to clarify a diagnosis and a prognosis. The vulnerability of the patient in this situation raises the question of whether his or her choice will be actively and freely made [8].

Moreover, a large number of people consider the information leaflet difficult to understand despite the short 2-page format [24]. The doctors surveyed admitted themselves that the majority of their patients did not read it. This question is related to health literacy issues [25–27].

Furthermore, the solicitation of consent by the doctor in charge of their health care calls into question the patient's autonomy to make this decision, even if a few of the doctors remind the patient that a refusal will have no impact on the care given. Despite these remarks, no major difference was found between those people directly or indirectly affected by cancer and the others.

#### The limitation of ethical issues to the patient's perception of physical risk

We have described how astonished some of our interviewees were when we asked them about their perception of the risks related to the repurposing of samples and their use in research and how their views changed over the course of the interview. These results suggest that, 'in real life', patients grant consent without truly understanding the issues at stake and without being familiar with ethical and scientific questions and regulatory frameworks. These same results are also found in the literature: 'Their willingness may reflect their un-informed naivete about the kind of research that might in future use their tissue sample' [19].

Thus, a large proportion of non-experts have only a vague idea of the types of research that will be carried out and very little knowledge of research ethics. Risk is essentially thought of in terms of 'physical risks' to the person, linked for example to the act of taking a sample. If there are acts that could potentially endanger the life or health of persons or constitute an attack on their bodily integrity, they are carried out for the sake of care, and therefore in the interest of the patient. The sample taken from the person cannot involve any risks since the research is carried out 'remotely'.

*As far as I know, if you work on a piece of a person's body that has been removed, it has no consequence on that person. (male, age 32, librarian, genetic predisposition)*

These representations are in line with a certain institutional vision historically built around the reflection on biomedical experiments. This reflection was initiated in the eighteenth century and especially in the nineteenth century [28], then reinforced and given substance in the twentieth century, when it became particularly focused on clinical trials. These representations do not correspond to current research practices, particularly in the context of translational research [29].

The ethical issues surrounding biobanks concern mainly two points: the purpose of the research, which is not yet known at the time consent is obtained, and data-sharing, a point that is not very clearly explained to the people contacted. It seems that one of the most problematic points is the lack of knowledge about the uses that could be made of their sample, making it impossible to envisage a limitation of certain uses. Studies show that once different uses that might be problematic for religious, moral or philosophical reasons are suggested, people tend to change their attitudes towards consent: 'They also have significant moral concerns about how their specimens might be used—even if they are unaware of such uses' [17].

Moreover, the participants' perception that granting consent carries a low level of risk is also largely linked to a lack of information, particularly on the potential sharing of data between researchers and between the database and teams outside the hospital. The context in which this consent is solicited presupposes (from the participants' point of view) that the sample will be used for no other purpose than research. A majority of French people trust their national research institutions and/or their doctor (that is, the one who solicits consent). They therefore consider that their sample will not be misused, a situation which they find difficult to imagine in any case. The question of trust is addressed in a few international publications [14, 30–32]. Their conclusions vary depending on national contexts, but the desire to participate generally decreases when it comes to sharing data with private pharmaceutical companies, whose primary aim is to make a profit [30, 33]. The people we interviewed were divided on this point: one group had reservations while another considered it an incontrovertible fact that the development of new drugs is essentially carried out by pharmaceutical companies. The latter group was mostly made up of the people we have called 'experts', some of them academics who work on data. They are better informed about the ethical risks and possible uses of samples, either in the field of research or not [34], although this would not affect their willingness to participate if asked to. Those who are themselves researchers are more sensitive to the need to have access to quality data, but also to a certain sense of solidarity, at least in our study population.

One of the risks mentioned in the literature [7] is that of stigmatisation and discrimination, e.g. a future employer or health-insurance provider gaining access to information on a person's current (or future, in the case of a genetic predisposition) health profile. That said, the experts in our study assumed that the samples and data would not be accessible for purposes other than research.

### Is granting consent an act of solidarity?

Studies show that people's choices are governed by different motivations. This analysis applies to the people in our study living in poverty, who imagine that if they were ill, they would draw benefits from giving their consent. They tend to overestimate those benefits, while at the same time underestimating the risks.

Conversely, the actual patients in our study population very rarely expect direct benefits from their contribution. People with genetic predispositions in general, and more specifically the participants in our study, expect more benefits for their children and for future generations. In the latter case, the benefit is therefore deferred, although it remains uncertain and applies to society as a whole. Some of the people we spoke to feel that consent was part of a cycle, that of *giving* to medicine because it makes scientific progress possible (and in particular, therapeutic progress to overcome cancer) and, on the part of certain patients, of *receiving* because they have benefited from therapeutic advances thanks to those who gave before them. This cycle, described by Mauss [35], describes a reciprocity that does not necessarily imply an equivalence between what is given and what is received. Rather, it is mediated through symbolic entities such as science and the hospital as an institution. Some of the people, especially those we have qualified as experts, feel that their consent makes sense within the framework of the French Social Contract, whereby the State guarantees access to health care and, in return, each citizen is expected to participate in research. This also implies that if commercial interests were put forward, people might be less inclined to participate.

However, some people grant their consent without expecting any individual or collective benefit. For them, it is an act devoid of any meaning, given the low value they attach to the sample.

#### Epistemological issues and their limits

The absence of precise information on the type of research to be carried out, limited here to cancer research, rarely constituted a difficulty or an obstacle to people's granting consent. However, this consent seemed to be based on a lack of awareness of the issues, which led to a change in their point of view as the interview progressed. This raised epistemological questions. Expectations emerged and then developed gradually in the course of the interview and as the participants came to a better understanding of the questions. This interview process is far from a real-life situation of granting consent. In most interviews, the aim was not to collect preconceived points of view—'people have no pre-existing knowledge or opinions about biobanking' [8]—but to provide different prompts that would encourage the participants to reflect on questions that some of them had never asked themselves before. According to Johnson et al. [36], the extent to which the arguments put forward accurately predict what people would actually do in real life is limited.

The researchers in this study became aware of these difficulties because the first interviews were sometimes punctuated by silence and hesitations, which corresponded to time needed for reflection. This was at times expressed clearly by the interviewees. Take, for example, the following woman who signed a consent form, but who was opposed to research on cancer drugs and was convinced that a body should be left to heal naturally. She agreed to undergo surgery to have her tumour removed but refused hormonal therapy.

*[Silence.] Listen, frankly, I've never thought about that. I admit that I don't know how to answer. Let's say that it's a question that hadn't occurred to me. Fine, Dr D asked me; fine, I consented, but I was thinking about research in hospitals, not necessarily in laboratories! (female, age 68, breast cancer)*

We were careful not to force a response when participants were at a loss and unable to formulate an opinion.

This also reveals the extent to which consent solicited in real life is not based on informed consent, since the dynamic interaction in an interview changes the participants' viewpoint as more detailed and in-depth information is provided.

Moreover, the results of this study on individuals' consent when donating samples to a tumour bank is a specific situation that cannot be generalised to other forms of participation in research. Indeed, biological samples were collected and health data gathered in the context of health care, without there being any additional physical constraints or risks related to their participation. And in some cases, this consent is inextricably linked to the tumor nature of sample, a waste product.

## Conclusion

In this analysed context of Carpem biobank, individuals, before they consent, are not aware of the precise objectives and purposes of the research that will be conducted. Therefore, they cannot commit themselves, and if they do, it is only in a vague way. So, consent is based on the motivation of individuals to participate in progress made in the field of medicine: they have in mind therapeutic perspectives (particularly in the case of cancer) rather than a personalised medical approach with potential direct benefits for themselves.

From an ethical point of view, providing information cannot be limited to a specific moment in time if consent is to be considered 'informed', given the lack of knowledge of the risks and issues involved. When consent is solicited, a certain amount of information is missing, even though we feel that more information would not alter consent or, if it did, would do so only marginally.

Given the fact that part of the act of consenting in France is based on the implicit trust that people have in the hospital soliciting it and in researchers in general, the lack of transparency with respect to the sharing of data with institutions outside the hospital is problematic. Transparency is also the ground on which trust rests for experts and non-experts alike. It would be unfortunate if consent were to be undermined by an erosion of trust that would be deleterious to future research practices and would induce biases if refusals were socially differentiated [31, 37].

People's low level of ethical culture and their lack of understanding of the scientific and ethical issues of biobanking research may well constitute a stumbling block in our study, but this does raise a more fundamental question: How can we make the greatest number of people aware of these issues and enable them to gain a better understanding of the challenges in a context where the complexity of research is ever increasing?

## Abbreviation

CARPEM: Cancer research for Personalized medicine

## Footnotes

1 We use the term 'representation' rather than 'knowledge' because for most people it is more an 'image belief' [14] than a true knowledge of the ethical rules governing research. The terms associated with 'representation' are *I think*, *I imagine* and not *I know that*…

2 This notion was introduced in 2008 in the Civil Code, Article 16-1-1: 'The respect due to the human body does not cease with death'.
